# The Use of Screen-Printed Electrodes in a Proof of Concept Electrochemical Estimation of Homocysteine and Glutathione in the Presence of Cysteine Using Catechol

**DOI:** 10.3390/s140610395

**Published:** 2014-06-12

**Authors:** Patricia T. Lee, Denise Lowinsohn, Richard G. Compton

**Affiliations:** 1 Department of Chemistry, Physical and Theoretical Chemistry Laboratory, Oxford University, South Parks Road, Oxford OX1 3QZ, UK; E-Mail: patricia.lee@chem.ox.ac.uk; 2 Department of Chemistry, Instituto de Ciências Exatas, Universidade Federal de Juiz de Fora, 36036-330 Juiz de Fora, MG, Brazil; E-Mail: denise.lowinsohn@ufjf.edu.br

**Keywords:** catechol, carbon nanotube screen printed electrodes, thiols, 1,4-Michael addition reaction, homocysteine, glutathione, cysteine, cyclic voltammetry

## Abstract

Screen printed electrodes were employed in a proof of concept determination of homocysteine and glutathione using electrochemically oxidized catechol via a 1,4-Michael addition reaction in the absence and presence of cysteine, and each other. Using cyclic voltammetry, the Michael reaction introduces a new adduct peak which is analytically useful in detecting thiols. The proposed procedure relies on the different rates of reaction of glutathione and homocysteine with oxidized catechol so that at fast voltage scan rates only homocysteine is detected in cyclic voltammetry. At slower scan rates, both glutathione and homocysteine are detected. The combination of the two sets of data provides quantification for homocysteine and glutathione. The presence of cysteine is shown not to interfere provided sufficient high concentrations of catechol are used. Calibration curves were determined for each homocysteine and glutathione detection; where the sensitivities are 0.019 μA·μM^−1^ and 0.0019 μA·μM^−1^ and limit of detections are *ca.* 1.2 μM and 0.11 μM for homocysteine and glutathione, respectively, within the linear range. This work presents results with potential and beneficial use in re-useable and/or disposable point-of-use sensors for biological and medical applications.

## 1. Introduction

Screen-printing technology is a well-known and established technology for mass producing electrodes [1–3]. The attractiveness of screen printing technology lies in the production process, for it makes electrodes fast, easy and simple to fabricate. A general screen-printed electrode can comprise any number of electrodes, from as few as one to a whole array of working electrodes on a supporting material with all the necessary integrated circuitry to satisfy the desired application [4]. Though there are some difficulties with commercializing screen-printed array electrodes, the market for the single working electrode is well established and is very advantageous as they are compact, low in cost, versatile, robust and disposable [1,4]. Especially for carbon-based electrodes, the inks used are usually comprised of graphite and/or carbon nanotube, so they have attributes similar to the conventional carbon-based electrodes of being low in cost, possessing a low background current and a wide window of working potentials [2,5]. To satisfy the growing technology of present and future applications, screen-printed electrodes are becoming more attractive and ideal for applications that require high throughput screening or do not require the need for complex and expensive equipment, such as bio-applications [1,2].

**Figure 1.**
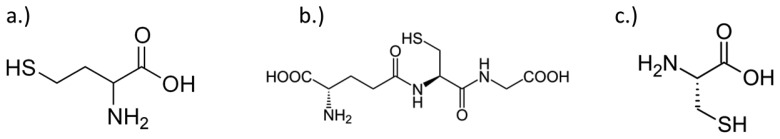
Chemical structure of thiols: (**a**) homocysteine; (**b**) glutathione; (**c**) cysteine.

Thiols such as homocysteine, HCys (Figure 1a), and glutathione, GSH (Figure 1b), are known to offer biological insight in respect of reflecting normal metabolism functions and homeostasis [6]. The levels of these antioxidants are becoming more important in clinical studies as reports have shown that elevated amounts may have implications for diseases such as cancer [7,8], atherosclerosis [6,7], cardiovascular disease [6,7,9], Parkinson's [6,10,11] and Alzheimer's disease [6,10,11]. Typically, homocysteine and glutathione can be found in physiological fluids, such as plasma and urine, where they can be valuable biomarkers for those studies [10–12]; the levels seen are normally within the ranges of 5.0–15 μM and 2.0–12 μM, respectively [6,9,11–17]. As such, monitoring these specific biomarkers can be a challenge. Assessment of these analytes is typically done with expensive instrumentation coupled with a separation technique, such as high performance liquid chromatography (HPLC) [9–11,14,18], liquid chromatography-mass spectroscopy (LC-MS) [9,19], gas chromatography-mass spectroscopy (GC-MS) [9,20] or liquid chromatography-ultraviolet spectrophotometry (LC-UV) [12,15]. These techniques can be expensive and complex in both operation and procedure, thus it would be highly desirable to have a simple, robust method for both biological and clinical studies [15]. Electrochemical detection methods can offer a number of advantages, such as being fast, facile and easy to perform. The literature reports the use of electrochemical methods for thiol detection, but unfortunately not many offer selectivity without the use of extensive sample pre-treatments [21–34], such as separation techniques [15,25]**,** or the addition of reagents to increase selectivity and/or to avoid other thiols from interfering in the measurement [32,35,36]. Currently, there are only limited literature reports on the selective detection of specific thiols (homocysteine, glutathione, and/or cysteine) in the presence of each other using voltammetric methods [32,37,38]. However, many of those methods require the use of an at least partially non-aqueous system. It would be highly desirable to provide a *simple* electrochemical method that provides the user with access to easy sample preparation with a unit that can possibly promote mobility for at part-of-use applications [31,32,37].

**Figure 2.**
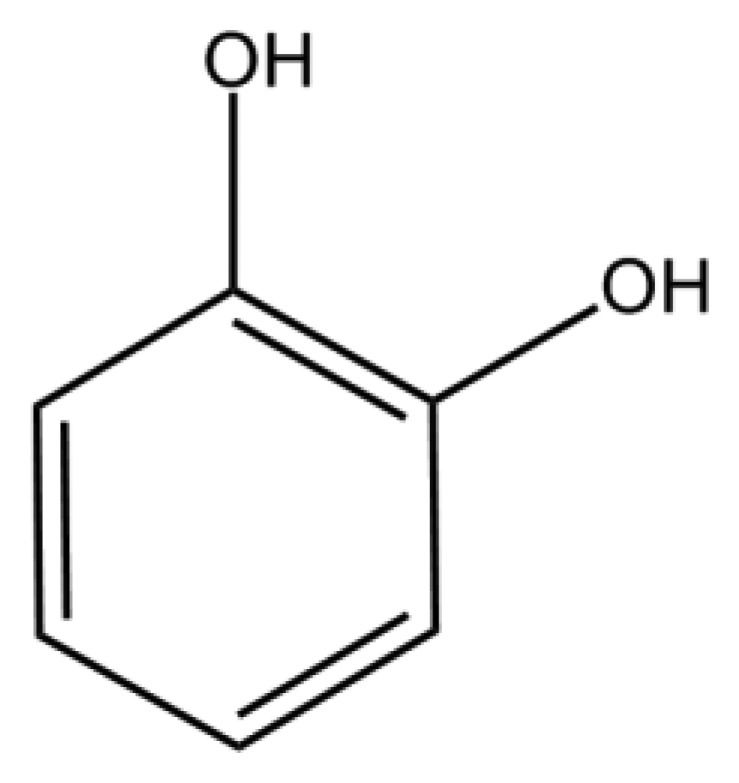
Chemical structure of catechol.

**Figure 3.**
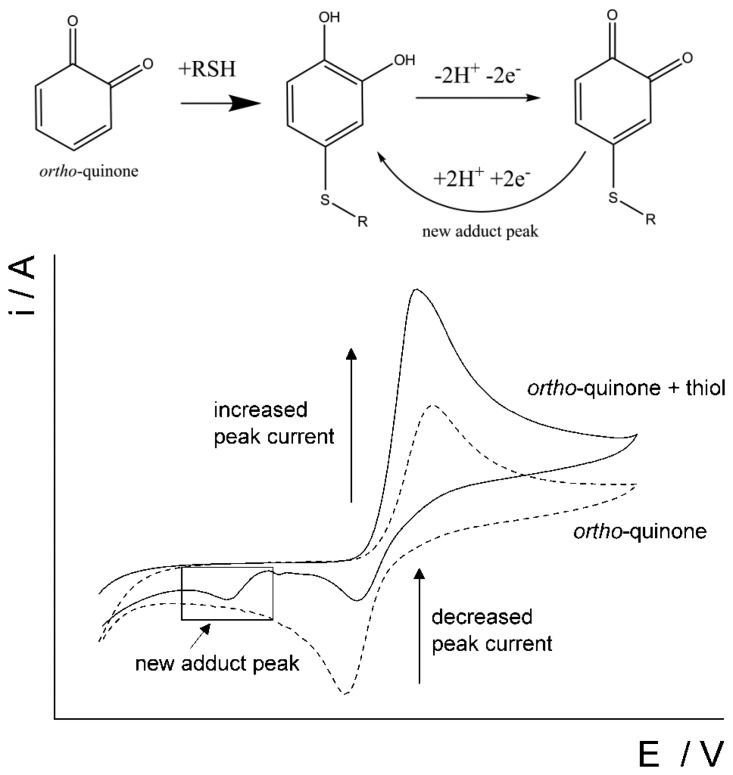
Schematic representation of the reaction between a thiol and *ortho*-quinone and the resulting voltammetric changes.

**Scheme 1.**
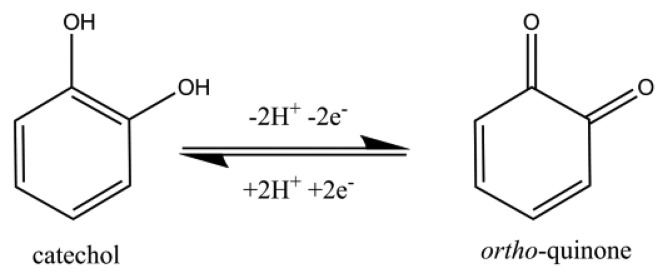
Oxidation of catechol to form *ortho*-quinone.

This paper reports on the use of disposable screen-printed electrodes to facilitate the determination of homocysteine and glutathione in the presence of each other in a pure aqueous system by utilizing an *ortho*-quinone as the mediator. There is some literature that reports the use of quinones to aid in the detection of thiols [16,23,39–44]. To the best of our knowledge, we are the first to report on the determination of homocysteine and glutathione in a pure aqueous system at a screen-printed electrode without the use of extensive sample pre-treatment. Catechol (CAT, Figure 2), was chosen to promote the 1,4-Michael addition reaction with thiols, RSH, [45,46] such as homocysteine and glutathione in the pure aqueous system. As the catechol is electrochemically oxidized to form an *ortho*-quinone (Scheme 1), then the *ortho*-quinone is susceptible to a nucleophilic attack by a thiol, RSH. As evidence of the 1,4-Michael addition reaction, the appearance of a new adduct peak coinciding with an increase in the forward peak and a decrease in the back peak is typically seen in voltammetry (Figure 3) [45,46]. The new adduct peak seen in the voltammogram is a result of the nucleophilic attack on the *ortho*-quinone by glutathione or homocysteine [7,45,47,48]. Cysteine does not typically produce a new signal [47,49]. Although the selective detection of homocysteine using electro-oxidized catechol in the presence of other thiols was previously reported [47], this work will expand on that notion by further investigating the proof of concept of determining the glutathione content in the same solution as homocysteine with a re-useable carbon electrode.

The proposed method essentially takes advantage of the different rates of reaction of homocysteine and/or glutathione with the electrochemically oxidized catechol using voltammetric methods. Allowing the faster reaction with homocysteine to initially take place by applying a high voltage scan rate, this makes it possible to “outrun” the slower reaction with glutathione resulting in the detection of an analytically useful adduct peak for measuring the amount of homocysteine. Thereafter, a slower scan rate is applied giving sufficient time for both analytes to react thus leading to a new adduct peak at low scan rate. The glutathione content can then be determined by subtracting the determined homocysteine value from the total adduct peak obtained at the slower scan rate. This proof of concept work was further applied to pre-determined mixed solutions containing both analytes (glutathione and homocysteine) and then later applied again with mixed solutions containing another added thiol compound, cysteine (Figure 1c) to further emphasize the value of this proposed procedure towards possible practical applications on physiological fluids (*i.e.*, urine and/or plasma). Carbon nanotube screen printed electrodes were used throughout this study. It has previously [47] been emphasized that the use of an electrode with a porous surface layer, such as a carbon nanotube-modified working electrode, promotes “thin-layer”-like diffusion [47,50–53] rather than semi-infinite diffusion. Under these diffusion conditions, the porous layer reduces the rate of transport of the thiols and therefore facilitates observation of the chemical reaction in the voltammetry. With conventional carbon electrodes (*i.e.*, glassy carbon electrode) that offer semi-infinite planar diffusion [47,51], the thiol reaction with *ortho*-quinone may be too slow for any useful observations to take place. In addition, the use of screen-printed electrodes continues to add upon the proposed idea of a possible portable sensor, whether it is for space-saving equipment in the lab or a point-of-care system for a medical facility.

## 2. Experimental Section

### 2.1. Reagents

All reagents their highest available purity were purchased through Sigma-Aldrich (Gillingham, UK) and Lancaster Synthesis (Lancaster, UK) and were used as received without any further purification steps; catechol (99%, Aldrich, Gillingham, UK), glutathione (98%, Sigma-Aldrich), D,L-homocysteine (≥95%, Sigma), and D,L-cysteine (97%, Lancaster Synthesis). All solutions were prepared with deionized water at a resistivity of no less than 18.2 MΩ·cm^−1^ at 25 °C (Millipore, Watford, UK). The buffer solutions, 0.15 M, were prepared using potassium monohydrogen phosphate (K_2_HPO_4_) (≥98%, Sigma-Aldrich), potassium dihydrogen phosphate (KH_2_PO_4_) (≥99%, Sigma-Aldrich), and potassium hydroxide (KOH) (≥85%, Sigma-Aldrich) accordingly to the required pH range. All buffer solutions were freshly made prior to experiments with supporting electrolyte of 0.10 M potassium chloride (KCl) (99%, Sigma-Aldrich) added to each solution.

### 2.2. Apparatus

**Figure 4.**
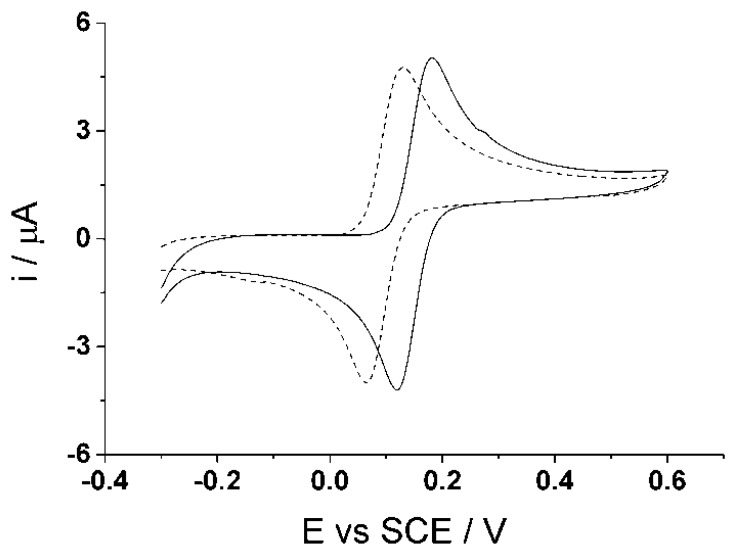
Cyclic voltammograms (50 mV·s^−1^) of screen printed electrode in 100 μM catechol using all three electrodes on the card.

The electrochemical experiments were carried out in a three electrode system using a saturated calomel electrode (SCE), reference electrode (Hach Lange, Salford, UK), a platinum mesh 99.99% (Goodfellow, Huntingdon, UK) counter electrode and multi-walled carbon nanotube disposable screen printed electrodes (DropSens, Llanera, Spain) as the working electrode. Figure 4 shows cyclic voltammograms (50 mV·s^−1^) recorded in 100 μM catechol (PBS, pH 7.0) solution using the reference and counter electrode provided on the screen printed electrodes.

The figure shows that the redox potential of catechol differs in the two voltammograms because the reference on the screen printed electrode is a silver pseudo reference. Therefore the purpose of using an external calomel reference electrode is to ensure that all potentials are the same in every condition. The distance among the electrodes were fixed to each other to ensure little to no change in the resistance. All experiments were conducted using a computer controlled potentiostat, PGSTAT 101 (ECO-chemie, Utrecht, Netherlands). A temperature controlled bath was also used to ensure that all electrochemical experiments were carried out at (20 ± 2) °C in a Faraday cage. All pH measurements were conducted using a pH213 Microprocessor pH meter (Hanna instruments, Leighton, UK). The pH meter was calibrated using Duracal buffers of pH 4.01 ± 0.01, pH 7.00 ± 0.01, and pH 10.01 ± 0.01 (Hamiliton, Bonaduz, Switzerland).

### 2.3. Carbon Nanotube Screen Printed Electrode (CNT-SPE)

Multi-walled carbon nanotube screen printed electrodes (CNT-SPE) were purchased from DropSens. The disposable screen printed electrodes are made of a ceramic substrate consisting of multi-walled carbon nanotube working electrode, a carbon counter electrode and a silver reference electrode. The surface area of the CNT-SPE was determined with a variable scan rate study in 1.0 × 10^−4^ M hexaammineruthenium (III) chloride and 0.1 M potassium chloride solution. Using the Randle-Ševčik equation, the determined average surface area is (0.11 ± 0.07) cm^2^.

**Figure 5.**
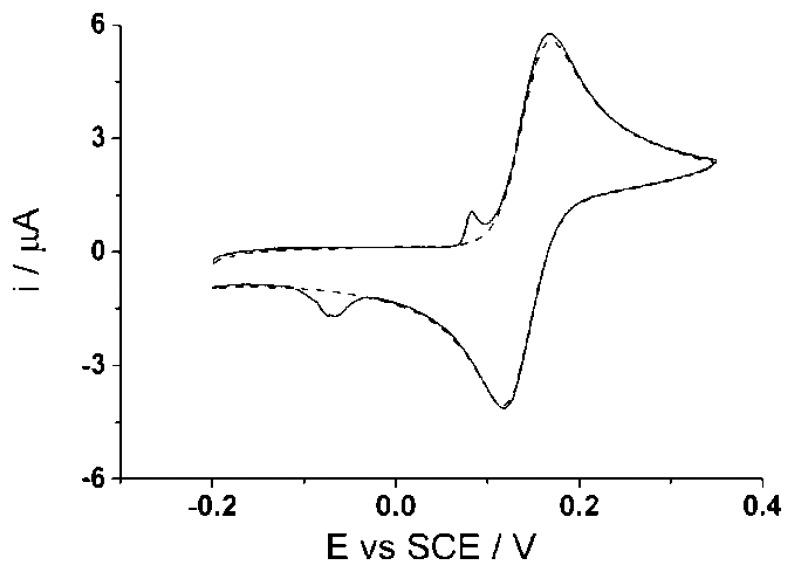
Cyclic voltammograms (50 mV·s^−1^) of screen-printed electrodes in 100 μM catechol (0.15 M PBS, pH 7.0, 20 °C) of before (solid lines) and after (dashed lines) electrochemical pre-treatment.

Prior to all electrochemical experiments, the CNT-SPE underwent a pre-treatment to get rid of any possible silver residues on the working electrode as supplied. The pre-treatment essentially comprises applying a potential cycling from −0.5 V to +0.2 V for an optimum time of 20 min (*ca.* 86 scans) in a solution of 0.1 M sodium nitrate with a mercury sulphate reference electrode. After pre-treatment, the screen printed electrode was carefully rinsed with de-ionized water and dried. Figure 5 shows a cyclic voltammogram (50 mV·s^−1^) comparison of the screen-printed electrode before (solid line) and after (dashed line) the pre-treatment in 100 μM catechol (PBS, pH 7.0).

The figure shows that before pre-treatment, the peaks at potentials *ca.* +0.1 V and −0.1 V (*vs.* SCE) are associated with the oxidation of silver to form silver chloride and its reverse reaction. After the pre-treatment is applied, the cyclic voltammogram shows no appearance of the peaks associated with possible silver. The lifetime and reproducibility of the electrode was observed with continual daily used over several months; cyclic voltammetry was applied in PBS (pH 7.0) at the end of each experiment to observe if any adsorption or contamination has occurred. Different electrodes from the same batch were also tested and show good reproducibility, ≥95%. It is recommended that the electrode be reused daily with careful rinsing and drying prior to the next experiment to prevent from any cross contamination and/or changes to the electrode area. Different electrodes from the supplier were tested to determine the reproducibility. The precision of the presented data will be representative of the intra-electrode.

## 3. Results and Discussion

### 3.1. Homocysteine Selectivity in the Presence of Glutathione

**Figure 6.**
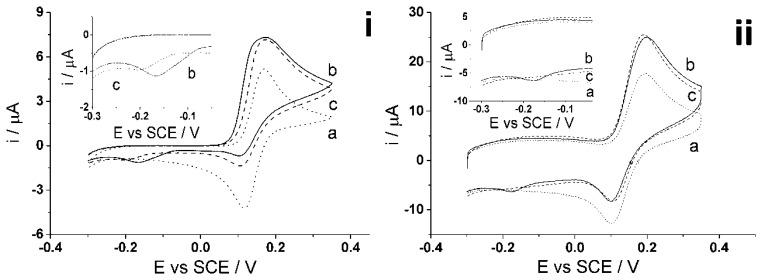
Cyclic voltammetry: (**i**) low scan rate (50 mV·s^−1^) and (**ii**) high scan rate (500 mV·s^−1^) of 100 μM catechol (PBS, pH 7.0 at 20 °C) (a) absence and presence of (b) 100 μM homocysteine (c) 100 μM glutathione (0.15 M PBS at 20 °C) at CNT-SPE. Inset: magnify view of adduct peaks.

The determination of homocysteine with catechol at a carbon nanotube modified electrode (CNT-GCE) was reported in an earlier paper [47]. Shown in the Figure 6ib, the cyclic voltammetric response of electro-oxidized catechol when homocysteine is present in solution shows an increase in the forward peak, decrease in the back peak and the appearance of a new adduct peak; thus indicating a 1,4-Michael addition reaction between homocysteine and oxidized catechol (shown above in Figure 3). Experiments were next carried out to investigate the determination of both homocysteine and glutathione using catechol solution at the CNT-SPE.

Initial results (Figure 6i) showed that upon applying a low scan rate using cyclic voltammetry (50 mV·s^−1^) to a solution containing 100 μM of each catechol (PBS, pH 7.0) (Figure 6ia), homocysteine (Figure 6ib) and glutathione (Figure 6ic), an adduct peak appears reflecting the oxidized catechol reaction with both glutathione and homocysteine, thus making it difficult for quantification when both are present in the solution. This is due to the slow scan rate used, allowing sufficient time for both homocysteine and glutathione to react with the electrochemically oxidized catechol. The new adduct peak observed in the voltammogram is due to the 1,4-Michael addition reaction, as described above, of *ortho*-quinone taking place with both glutathione and homocysteine at the electrode. This simple electrochemical behaviour at the CNT-SPE presents a problem with quantifying either homocysteine or glutathione in the presence of each other. Accordingly, the following two-step procedure is proposed.

**Figure 7.**
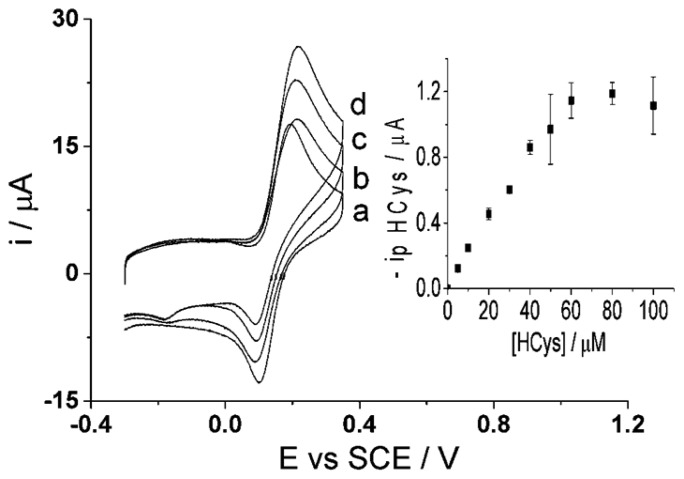
Cyclic voltammograms (500 mV·s^−1^) of 100 μM catechol (PBS, pH 7.0 at 20 °C) at varying homocysteine concentrations, (a) 0 M (b) 20 μM (c) 60 μM and (d) 100 μM, at CNT-SPE. Inset: Peak current of homocysteine adduct peak *vs.* concentration of homocysteine.

First, homocysteine selectivity was investigated by examining the reaction rates of glutathione and homocysteine with electro-oxidized catechol at the CNT-SPE. A higher voltage scan rate was applied with the aim to “outrun” the glutathione-catechol reaction but still allow the homocysteine-catechol reaction to take place. The optimized scan rate of 500 mV·s^−1^ [47] was applied using cyclic voltammetry of a solution containing 100 μM of each catechol, glutathione and/or homocysteine (PBS, pH 7.0) using the CNT-SPE (Figure 6ii). The results show little to no product peak appearance when the high scan rate is applied to a solution containing glutathione while an analytically useful adduct peak appears in the presence of homocysteine. This suggests that the higher scan rate was fast enough to preclude the catechol reaction with glutathione but still allow the reaction to take place with homocysteine thus allowing selective determination of homocysteine to also take place at CNT-SPE. Thus using 100 μM of catechol (PBS, pH 7.0), an analytical curve was obtained for homocysteine (Figure 7) as, I (μA) = (0.020 ± 0.000076) [HCys/μM] (*n* = 3), at homocysteine concentrations up to 60 μM. This measurement at high scan rate thus allows the selective measurement of homocysteine in the presence of glutathione at the screen printed electrode.

### 3.2. Determination of Glutathione in the Presence of Homocysteine

**Figure 8.**
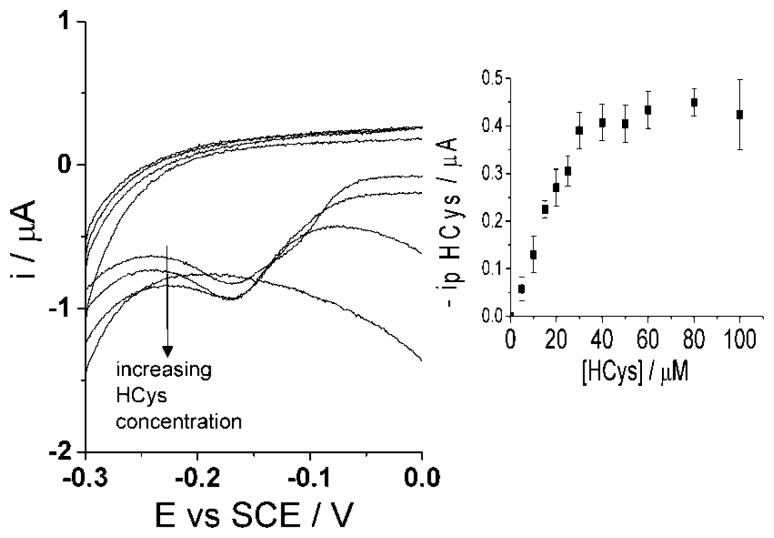
Cyclic voltammograms (50 mV·s^−1^) of adduct peak of 100 μM catechol (PBS, pH 7.0 at 20 °C) at varying homocysteine concentrations, 0–100 μM, at CNT-SPE. Inset: Peak current of homocysteine adduct peak *vs.* concentration of homocysteine.

**Figure 9.**
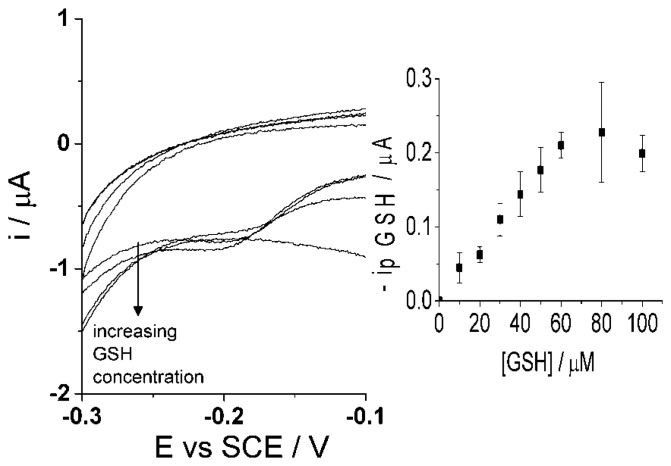
Cyclic voltammograms (50 mV·s^−1^) of adduct peak of 100 μM catechol (PBS, pH 7.0 at 20 °C) at varying glutathione concentrations, 0–100 μM, at CNT-SPE. Inset: Peak current of glutathione adduct peak *vs.* concentration of glutathione.

Though the selective detection of homocysteine is achievable at high scan rate, 500 mV·s^−1^, the concept of glutathione determination within the same sample is proposed in the following as the second step procedure. The same sample solution is agitated and then a low scan rate of 50 mV·s^−1^ [47] is applied using cyclic voltammetry. As discussed above, the low scan rate allows the reaction of both homocysteine and glutathione to take place with the electro-oxidized catechol thus resulting in the same peak potential for the adduct peak; consequently, the adduct peak will represent the contents of both analytes (Figure 6i). By this means, homocysteine is being measured at both scan rates and the adduct peak of pure homocysteine can then be subtracted from the total adduct peak obtained at low scan rate, containing HCys and GSH, thus resulting in a concentration of glutathione alone. Furthermore, calibration curves need to be obtained at the low scan rate, 50 mV·s^−1^, for each homocysteine and glutathione in the presence of 100 μM catechol (PBS, pH 7.0) using cyclic voltammetry. First, Figure 8 indicates that the calibration curve for homocysteine at a scan rate of 50 mV·s^−1^ shows a linear relationship, I (μA) = (0.013 ± 0.00013) [HCys/μM], with a linear range of 0–30 μM. Second, Figure 9 shows another calibration curve at low scan rate of 100 μM catechol (PBS, pH 7.0) at varying concentrations of glutathione, 0–100 μM. The figure also shows that peak current is proportional to concentration at the low scan rate, I (μA) = (0.0035 ± 0.000066) (GSH/μM) with a linear range from 0 μM to 60 μM of glutathione. Once all the appropriate calibration curves are determined, the proposed method can be applied and tested.

A preliminary experiment using a number of pre-determined mixed solutions with varying amounts of each homocysteine and glutathione, ranging from 1.0 μM to 10 μM, with 100 μM catechol (PBS, pH 7.0 at 20 °C) using CNT-SPE was carried out using the following two-step procedure. First, the concentration of homocysteine present in solution was determined using the peak current at high scan rate, 500 mV·s^−1^, using the analytical curve at this scan rate mentioned in Section 3.1, I (μA) = (0.020 ± 0.000076) [HCys/μM] at the CNT-SPE. Next, knowing the homocysteine concentration, using a different corresponding linear relationship for homocysteine, we calculate the peak current at a lower scan rate, 50 mV·s^−1^, I (μA) = (0.013 ± 0.00013) (HCys/μM) (Figure 8). We can subtract this value from the total adduct peak current at the low scan rate thus providing the current for glutathione-catechol reaction. The glutathione concentration can then be determined using the corresponding calibration curve at 50 mV·s^−1^, I (μA) = (0.0035 ± 0.000066) (GSH/μM) (Figure 9). Accordingly, the results can be seen in the Table 1, where most of the determined analyte values correspond to the real mix. At low concentrations, such as 1.0 μM homocysteine, there were difficulties measuring the correct peak current due to the peak current for the adduct peak being very small and thus hard to evaluate. Nonetheless, these results initially show that this procedure provides good agreement with the real mixture at an average detection standard deviation *ca.* 23% as the estimated value is compared to the real values in the mixture. The proposed method will work generally providing both glutathione and homocysteine are of much lower concentration then the added catechol.

### 3.3. Selective Determination of Homocysteine and Glutathione in the Presence of Cysteine

**Figure 10.**
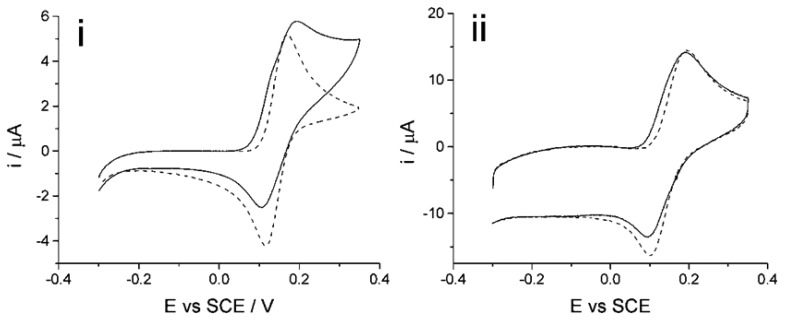
Cyclic voltammogram (**i**) 50 mV·s^−1^ and (**ii**) 500 mV·s^−1^ of 100 μM catechol in the absence (dotted line) and presence (solid line) of 30 μM cysteine (PBS, pH 7.0 at 20 °C) at CNT-SPE.

As mentioned above, we were able to show the viability of a trusted procedure for estimating both glutathione and homocysteine in the presence of each other. However, cysteine is another thiol that can also be present in biological media and can act as a possible interferrent since it is a homologue to homocysteine. To investigate whether cysteine interferes with the electrochemical measurement in the proposed method, an experiment was carried out at the CNT-SPE in a solution containing 100 μM catechol (PBS, pH 7.0, at 20 °C) and 30 μM cysteine using cyclic voltammetry (50 mV·s^−1^ and 500 mV·s^−1^). The concentration of 30 μM cysteine was used as reports have shown that 30 μM of cysteine can be a higher limit observed with certain body fluids such as urine [11,54,55]. Figure 10 shows the voltammograms of the electro-oxidized catechol in the absence (dotted line) and presence of cysteine (solid line) at scan rates 50 mV·s^−1^ (Figure 10i) and 500 mV·s^−1^ (Figure 10ii). The figures show that in the presence of cysteine at either scan rate, the forward peak increases while the back peak decreases but show no evidence of a new adduct peak. This suggests that the reaction between the electrochemically oxidized catechol and cysteine is not a 1,4-Michael addition but rather an electrocatalytic reaction [44,46] and will not interfere with the signal for homocysteine and/or glutathione provided the concentration of cysteine is low compared to that of electro-oxidized catechol. Thus this method using CNT-SPE can enable the possible determination of homocysteine and glutathione in the presence of each other and cysteine.

To further validate the proposed method, experiments were performed just as the described above in the presence of 30 μM cysteine. Calibration curves were determined using cyclic voltammetry for homocysteine (scan rates at 500 mV·s^−1^ and 50 mV·s^−1^) and glutathione (scan rate at 50 mV·s^−1^) all in the presence of 30 μM cysteine and 100 μM catechol (PBS, pH 7.0 at 20 °C), summarized in Table 2. The linear relationship of homocysteine at the high scan rate is I HCys at 500 mV·s^−1^ (μA) = (0.019 ± 0.00029) [HCys/μM] with a linear range of 0–40 μM. The linear range and the sensitivity of homocysteine detection at the high scan rate are relatively close when compared to values in the absence of cysteine. This suggests that there is no significant interference during the detection of homocysteine at high scan rate while in the presence of both cysteine and/or glutathione, provided the catechol concentration is sufficiently high. Next, the homocysteine relationship at the lower scan rate is, I HCys at 50 mV·s^−1^ (μA) = (0.077 ± 0.00029) [HCys/μM] with a linear range of 0–25 μM. The linear range and sensitivity for this case at the low scan rate is slightly lower when compared to the absence of cysteine. This is rationalized again provided that the concentration of the catechol is higher than the amount of thiol present in solution. Lastly, the linear relationship for glutathione at the low scan rate is, I GSH at 50 mV·s^−1^ (μA) = (0.0019 ± 0.000047) [GSH/μM] with a linear range of 0–60 μM. The sensitivity for glutathione detection in the presence of cysteine is also lower than in the absence; where similar to the detection of homocysteine at low scan rate; again, more catechol can be added to increase those values. The limit of detection (LOD) for this proposed method was determined using the following, 3*SD*/*S* where *SD* is the standard deviation given in the presence of zero analyte and *S* is the sensitivity, given by the slope of the calibration curve. In the presence of 30 μM cysteine, the LOD for homocysteine at the high scan rate is *ca.* 1.2 μM and since the determination of glutathione is dependent on the homocysteine detection, the LOD was determined for homocysteine at the low scan rate, therefore the LOD is *ca.* 0.11 μM. The determined LOD to the respected analytes are all within a reasonable range of detection needed for biological samples.

An experiment was carried out, similar to the preliminary test described in Section 3.2, but with the addition of 30 μM cysteine to further validate this method in solutions containing all three thiol analytes. A number of pre-determined mix solutions containing different quantities of homocysteine and glutathione, varied from 1.0 μM to 10 μM, were combined with a constant amount of catechol (100 μM, PBS, pH 7.0) and cysteine (30 μM, PBS, pH 7.0); thereafter, the proposed method described above was applied using CNT-SPE.

The results are then summarized in Table 3 where the values are within reasonable deviation, *ca.* 28%, from the pre-determined quantities present in the solutions. It should be noted that there were difficulties with quantification, especially at 1.0 μM homocysteine concentration, due to the low peak current produced at the high scan rate and confirms with the calculated LOD value of the system. Nevertheless, the values obtained in this test are within reason to match with the real contents of the mixture.

## 4. Conclusions

The electrochemical estimation of homocysteine and glutathione was shown to be achievable in the absence and presence of cysteine using the proposed two-step procedure at a commercial carbon nanotube screen-printed electrode. This method takes advantage of the different reaction rates of homocysteine and glutathione with the electrochemically oxidized catechol taking place at the surface of the screen printed working electrode via a 1,4-Michael addition reaction. It was shown that this method can be easily adaptable to real world applications due to its low limit of detection within a reasonable range seen in real samples. In addition, the proof of concept method was further proven to be successful as the determined values of homocysteine and glutathione were comparable to the real mixed sample solution contents determined using the two-step method. An average standard deviation of *ca.* 28% of the determined values was obtained when evaluated with the real contents in mixed solutions containing cysteine. This value, which derives from the ten-fold difference in sensitivity towards glutathione and homocysteine, is sufficient for medical applications where threshold values are typically required as “biomarker” signals. This easy two-step procedure involves little to no extensive sample pre-treatment to the user, which enables possible real-world applications in healthcare or medical research.

## Figures and Tables

**Table 1.** Determination of homocysteine and glutathione in mixed solution containing homocysteine, glutathione and 100 μM catechol (PBS, pH 7.0 at 20 °C).

Mixed Solution		Determined [Homocysteine] * (μM)	Determined [Glutathione] * (μM)

Homocysteine (μM)	Glutathione (μM)		
10	10	10.0 ± 0.0100	9.83 ± 0.230
5	10	5.1 ± 0.35	8.90 ± 0.850
10	5	9.4 ± 0.10	5.75 ± 0.150
10	3	9.3 ± 0.10	2.9 ± 0.20
3	10	3.3 ± 0.010	10.1 ± 0.650
1	10	1.6 ± 0.10	11.5 ± 0.250
10	1	10.3 ± 0.600	0.84 ± 0.01

* Average value ± standard deviation (*n* = 2).

**Table 2.** Summary of values employed for calculation to determine homocysteine and glutathione in the presence of catechol and cysteine.

Analyte	Slope (μA·μM^−1^)	y-Intercept (μA)	Regression Coefficient	Linear Range ** (μM)	Limit of Detection (μM)
Homocysteine at 500 mV·s^−1^	0.019	0	0.998	0–40 (8)	1.2
Homocysteine at 50 mV·s^−1^	0.077	0	0.997	0–25 (6)	0.11
Glutathione at 50 mV·s^−1^	0.0019	0	0.995	0–60 (7)	0.11

** linear range value (number of points employed for the calculation).

**Table 3.** Determination of homocysteine and glutathione in mixed solution containing.

Mixed Solution		Determined [Homocysteine] * (μM)	Determined [Glutathione] * (μM)

Homocysteine (μM)	Glutathione (μM)		
10	10	10.5 ± 0.850	11.4 ± 0.050
5	10	5.8 ± 0.35	9.7 ± 0.40
10	5	11.1 ± 0.150	4.6 ± 0.10
10	3	10.2 ± 0.250	3.2 ± 0.65
3	10	3.6 ± 0.15	10.2 ± 1.14
1	10	2.1 ± 0.15	10.4 ± 0.500
10	1	10.8 ± 0.400	1.3 ± 0.80

* Average value ± standard deviation (*n* = 2).
